# Influence of permanent pacemaker implantation after transcatheter aortic valve implantation with new-generation devices

**DOI:** 10.1007/s12471-018-1194-1

**Published:** 2018-11-15

**Authors:** B. Gonska, M. Keßler, J. Wöhrle, W. Rottbauer, J. Seeger

**Affiliations:** Department of Internal Medicine II, University of Ulm, Ulm, Germany

**Keywords:** Transcatheter aortic valve implantation, Permanent pacemaker implantation, Mortality

## Abstract

**Objective:**

Permanent pacemaker implantation (PPMI) after transcatheter aortic valve implantation (TAVI) is the most common complication after the procedure. PPMI rates remain high with the new-generation TAVI devices despite improved outcomes concerning paravalvular aortic regurgitation and vascular access complications. However, the impact of PPMI on mortality and clinical outcome is still a matter of debate, and data with new-generation devices on this matter are scarce. Therefore, we sought to analyse the influence of PPMI in patients treated with the new-generation devices on one-year outcome.

**Methods:**

We enrolled 612 consecutive patients without prior pacemaker undergoing transfemoral TAVI with the new-generation devices. Patients with or without PPMI were compared with respect to clinical outcome within one year.

**Results:**

PPMI was performed in 168 patients (24.4% of the overall study population). There was no significant difference in one-year outcome concerning all-cause mortality (PPMI vs. no-PPMI: 12.2% vs. 12.5%, *p* = 0.94), rate of major adverse events including cardiac, cerebral or valve-related events and bleeding complications (22.1% vs. 24.5%, *p* = 0.55) or need for rehospitalisation due to cardiac symptoms (16.1% vs. 18.1%, *p* = 0.63). In patients with reduced ejection fraction (<45%) there was also no impact of PPMI on one-year mortality (14.3% vs. 15.7%, *p* = 0.86). Furthermore, multivariate analysis did not reveal PPMI to be independently associated with one-year mortality (odds ratio 0.94, 95% confidence interval 0.50–1.74, *p* = 0.83).

**Conclusions:**

In this large all-comers TAVI population with new-generation devices the need for postprocedural PPMI did not show a statistical significant impact on survival or combined endpoint of major adverse events within one year.

## What’s new?

Data on the impact of permanent pacemaker implantation after TAVI with the new-generation devices are rare. Available follow-ups on this matter are from patients treated with the early-generation devices. Since survival after TAVI seems to continue to improve with the new-generation devices, but pacemaker implantation rate does not decline, the impact of pacemaker implantation in these patients needs to be further evaluated. We add first one-year follow-up data on outcome after TAVI in patients treated with the new-generation devices, which did not show a disadvantage in outcome for patients with permanent pacemaker implantation.

## Introduction

Within the last decade, transcatheter aortic valve implantation (TAVI) has emerged as the standard care for severe aortic stenosis in inoperable patients or in patients at high risk for surgical valve replacement (SAVR) [1, 2]. In patients at intermediate risk for SAVR, noninferiority of TAVI in comparison with SAVR was shown for the balloon-expandable Edwards Sapien valves (Edwards Lifesciences, Irvine, CA, USA) in the PARTNER 2A randomised trial [3] as well as for the self-expandable Medtronic CoreValve (Medtronic, Minneapolis, MN, USA) in the SURTAVI trial [4]. However, rate of permanent pacemaker implantation (PPMI) after TAVI is significantly higher compared with SAVR [5]. Even though the new-generation transcatheter heart valves have managed to reduce periprocedural complications, such as moderate or severe paravalvular aortic regurgitation or vascular access complications, when compared with the early-generation devices [6], PPMI still remains a common postprocedural complication, with tendencies to higher rates of PPMI compared with the early-generation transcatheter aortic heart valves [7, 8].

Rates of PPMI range from ~17% for the balloon-expandable Edwards Sapien 3 valve (ES3) [5], ~22% for the self-expandable Medtronic CoreValve Evolut R [9] up to 40% for the mechanically expanded Boston Lotus valve (Boston Scientific Corporation, Marlborough, MA) [10]. The impact of PPMI on further patient survival and quality of life is still a matter of debate. There is evidence of an association of chronic right ventricular pacing with the occurrence of heart failure in non-TAVI patients based on the MOST trial and DAVID trial, which is feared to be translated to the outcome of elderly patients with PPMI after TAVI [11, 12]. Data from the FRANCE-2 registry evaluating TAVI revealed PPMI after TAVI as an independent predictor for 3‑year mortality (*p* = 0.02) [13] , whereas a recently published meta-analysis of 23 studies including 20,287 patients undergoing TAVI did not observe an increased mortality during follow-up [14]. All of these data were generated from studies with the early-generation TAVI devices. Currently, there are no data on the impact of PPMI after TAVI with the new-generation devices.

Therefore, we wanted to analyse the influence of PPMI after TAVI with new-generation devices on mortality as well as on clinical outcome in a large single-centre all-comers patient collective within a 12-month period.

## Methods

We evaluated 688 consecutive patients undergoing transfemoral TAVI for treatment of symptomatic aortic stenosis (Clinical Trial Registration: NCT02162069). TAVI was performed at the University of Ulm (Germany) between February 2014 and September 2016. Baseline characteristics of patients with (PPMI) and without need for PPMI (no-PPMI) after TAVI were compared as well as clinical outcome within a 12-month period. Patients with pre-existing permanent pacemakers were excluded. Severe aortic stenosis was documented by echocardiography and cardiac catheterisation with an aortic valve area (AVA) ≤1 cm^2^ or an indexed AVA ≤0.6 cm^2^. Patients were at high or intermediate risk for SAVR indicated by the Society of Thoracic Surgeons (STS) score for mortality, were at high risk according to the risk assessment combining STS score for mortality, frailty or major organ system dysfunction according to the 2014 American College of Cardiology/American Heart Association Guidelines for Valvular Heart Disease [15] or had relevant further comorbidities favouring a transcatheter approach. There was a Heart Team decision for a transcatheter valve replacement in all patients. Written consent was obtained in all patients. The study was approved by the local ethics committee. All TAVI procedures were performed via a transfemoral approach under conscious sedation as described elsewhere [16, 17]. Predilatation was performed in all patients. The new-generation TAVI devices used were the balloon-expandable ES3, the mechanically expanded Lotus valve and the self-expandable CoreValve Evolut R. The size of the implanted valve was determined by a pre-procedural 256 multislice computed tomography as described elsewhere [18].

Primary endpoint was all-cause mortality at one year followed by a combined endpoint of major adverse events including all-cause-death, cardiac events (myocardial infarction, new onset of atrial fibrillation, heart failure), valve function related symptoms, stroke/transitory ischaemic attack, bleeding complications or vascular complications occurring after hospital discharge according to the Valve Academic Research Consortium 2 (VARC-2) criteria [19]. Furthermore, we evaluated the rate of rehospitalisation due to cardiac symptoms.

### Statistical analysis

We carried out statistical analysis with Statistica software version 10 (TIBCO Software Inc., Palo Alto, CA, USA). Continuous variables are expressed as mean ± one standard deviation and were compared with the T test. Categorical variables are presented as counts and percentages and differences between proportions were calculated by using the Chi-squared test. A *p*-value of <0.05 was considered statistically significant. Multivariate logistic regression analysis was carried out to identify independent predictors for all-cause mortality, including the following variables: PPMI, STS score for mortality >6.5%, left ventricular ejection fraction (LVEF) <45%, diabetes mellitus, valve type (including ES3 and Boston Lotus valve) and history of atrial fibrillation.

## Results

After excluding patients with a permanent pacemaker prior to the TAVI procedure, we carried out the analysis on 612 patients. PPMI after TAVI was needed in 168 patients (27.5% of patients without prior PPMI and 24.4% of the total patient cohort). Pacemaker specific details are displayed in Tab. 1.

**Table 1:** Pacemaker implantation indication and device type

	Number of patients
*Pacemaker indication*	
AVB I° with severely prolonged QT-duration and complete left bundle branch block	13 (7.7%)
AVB II° type Mobitz II	8 (4.8%)
AVB III°	118 (70.2%)
Trifascicular block	4 (2.4%)
Alternating complete right and left bundle branch block	6 (3.6%)
Bradyarrhythmia with atrial fibrillation	17 (10.1%)
Sinus arrest	1 (0.6%)
Resuscitation due to ventricular fibrillation	1 (0.6%)
*Device type*	
VVI-Pacemaker	34 (20.2%)
DDD-Pacemaker	123 (73.2%)
Implantable cardioverter defibrillator (2-chamber)	2 (1.2%)
Cardiac resynchronisation therapy—pacemaker	9 (3.0%)
Cardiac resynchronisation therapy—defibrillator	4 (2.3%)

*AVB* atrioventicular block

Mean population age was 80.4 ± 5.9 years. More than three quarters of patients were severely symptomatic with NYHA class III or IV (77.9%). The STS score for predicted risk of mortality of the cohort was 6.5 ± 4.9%. Mean aortic valve area acquired by echocardiography was 0.77 ± 0.22 cm^2^, mean aortic peak gradient 66.2 ± 23.5 mm Hg, mean LVEF 57.5 ± 15.2%.

Baseline clinical characteristics of patients, according to the pacemaker status after TAVI, did not show any statistically significant differences (Tab. 2). However, there was a significant difference in pre-existing conduction disorders between patients with and patients without PPMI after TAVI. The rate of a pre-existing first-degree atrioventricular block was significantly higher in patients with PPMI after TAVI (30.0% vs. 11.8%, *p* < 0.001) The same applies to the rate of a pre-existing complete right bundle branch block (22.2% vs. 2.9%, *p* < 0.001). Rate of complete left bundle branch block at baseline (10.2% vs. 10.5%, *p* = 0.94) did not differ between groups, neither did history of atrial fibrillation (39.3% vs. 34.3%, *p* = 0.30).

**Table 2:** Baseline clinical characteristics

	No-PPMI	PPMI	*P*-Value
Number of patients	444	168	
Age, years	80.1 ± 6.1	81.1 ± 5.5	0.08
Female	244 (55.0%)	80 (47.6%)	0.11
BMI (kg/m^2^)	26.9 ± 4.6	27.2 ± 5.0	0.48
NYHA functional class III/IV	350 (81.1%)	127 (75.6%)	0.13
Diabetes mellitus	140 (31.5%)	43 (25.6%)	0.15
Severe chronic renal failure	43 (9.7%)	20 (11.9%)	0.42
Coronary artery disease	268 (60.8%)	106 (63.5%)	0.54
History of myocardial infarction	60 (13.5%)	24 (14.3%)	0.80
History of cardiac surgery	47 (10.6%)	15 (8.9%)	0.64
History of stroke or intracerebral bleeding	45 (10.1%)	18 (10.7%)	0.83
Pulmonary disease (moderate or severe)	189 (42.8%)	78 (46.7%)	0.38
History of atrial fibrillation	154 (34.7%)	66 (39.3%)	0.30
EuroSCORE II, %	6.2 ± 5.8	6.3 ± 5.5	0.84
STS PROM, %	6.6 ± 4.8	6.7 ± 5.2	0.89
*Echocardiographic data*			
Aortic peak gradient, mm Hg	64.1 ± 22.4	66.1 ± 23.3	0.33
AVA indexed, cm^2^/m^2^	0.28 ± 0.08	0.28 ± 0.08	0.54
Left ventricular ejection fraction (%)	57.1 ± 15.4	58.0 ± 14.7	0.51
Left ventricular ejection fraction <45%	94 (21.2%)	30 (17.9%)	0.36
*Procedural data*			
Implanted valve type:			
– Boston Lotus/Lotus Edge	127 (28.6%)	98 (58.3%)	<0.01
– Edwards Sapien 3	294 (66.2%)	66 (39.3%)	<0.01
– Medtronic CoreValve Evolut	23 (5.2%)	4 (2.4%)	0.13
Paravalvular aortic regurgitation			
– None/trace	343 (77.3%)	129 (82.7%)	0.14
– Mild	101 (22.8%)	29 (17.3%)	
– Moderate/severe	0	0	
Device success	410 (92.3%)	154 (91.7%)	0.78

*BMI* body mass index, *NYHA* New York Heart Association, *STS PROM* Society of Thoracic Surgeons Score for predicted risk of mortality, *AVA* aortic valve area

Implanted valves were the ES3 valve in 360 patients (58.8%), the Boston Lotus valve in 218 patients (35.6%), the Boston Lotus Edge valve in 7 patients (1.1%) and the Metronic CoreValve Evolut R in 27 patients (4.4%). In patients with PPMI after TAVI, the Boston Lotus valve had the highest proportion within the valve distribution, whereas the ES3 valve had the highest proportion in the patients without PPMI after TAVI.

Device success according to the VARC-2 criteria was high with 92.2%. There was no periprocedural death within the first 72 hours after the procedure. Five patients died within the initial hospital stay between the 20th and 51st day after TAVI. Intrahospital rate of stroke or transient ischaemic attack was 2.5% for no-PPMI and 5.4% for PPMI patients without statistical relevance (*p* = 0.07).

Major or life-threatening bleeding complications after TAVI according to the VARC-2 criteria were significantly higher in patients after PPMI (8.9% vs. 3.8%, *p* = 0.01), driven by a significantly higher rate of major bleeding events (5.4% vs. 2.5%, *p* = 0.01). However, the higher proportion of patients with major bleedings events did not occur due to the PPMI (only 2 patients of all patients with major bleeding events). Rate of life-threatening bleeding events was low with 3.0% (PPMI) and 1.8% (no-PPMI) (*p* = 0.37), with 3 patients having a pericardial effusion after PPMI. Further complications after PPMI were re-operation due to lead dislocation in 3 patients and haematoma/bleeding event at the site of the pacemaker in 4 patients. There was no pneumothorax. Ventricular pacing burden at pacemaker check 1 to 4 days after the implantation was 68.0 ± 43.3% (median 98%, interquartile range [IQR] 11–100%), with 59.9% of patients with a ventricular pacing burden ≥90% and 8.1% of patients with a ventricular pacing burden of 0%.

### Outcome at thirty days

Follow-up rate was 99.7%. We could not obtain 30-day follow-up in two patients (both patients without PPMI after TAVI). For the remaining 610 patients, clinical outcome did not show a significant difference between patients with or without PPMI post TAVI. Data are displayed in Tab. 3. Rate of mortality was low with only 1.1% in the group of patients without PPMI and 1.8% in the group of patients with PPMI (*p* = 0.53). The rate of the combined endpoint of major adverse events since hospital discharge after TAVI again was not distributed differently between groups (4.3% no-PPMI vs. 4.2% PPMI, *p* = 0.95). There was also no significant difference in the individual events from the combined endpoint between groups. Ventricular pacing burden had decreased to a mean of 39.3 ± 43.6%, (median 11%, IQR 11–98%) with 30.9% of patients with a pacing burden of ≥90% and 17.1% of patients with a pacing burden of 0% (data available on ventricular pacing burden for 73% of patients).

**Table 3:** Clinical outcome at 30 days

	No-PPMI	PPMI	*P*-Value
Number of patients	442	168	
Major adverse events	4.3%	4.2%	0.95
All-cause mortality	1.1%	1.8%	0.53
Stroke or transient ischaemic attack	1.4%	0.6%	0.43
– Stroke	0.2%	0.6%	0.47
– Transient ischaemic attack	1.1%	0%	0.17
Myocardial infarction	0%	0%	–
Bleeding	0.5%	0.6%	0.82
Aortic dissection	0%	0.8%	0.10
Aortic valve thrombosis or endocarditis	0%	0%	–
New onset of atrial fibrillation	0%	0%	–
Rehospitalisation	2.8%	1.8%	0.51

### Outcome at one year

**Fig. 1:**
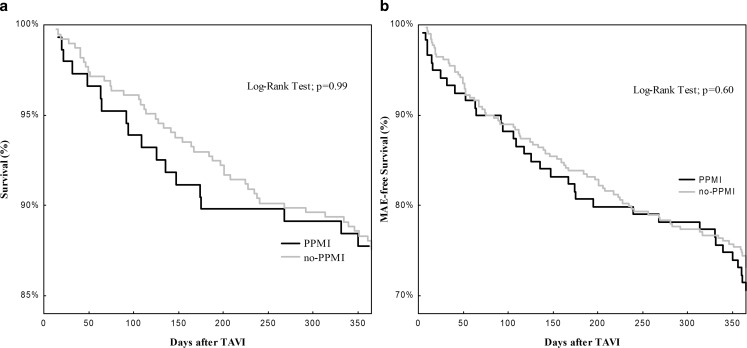
Kaplan-Meier curves for survival with respect to pacemaker status after TAVI (**a**) and MAE-free survival with respect to pacemaker status (**b**). *TAVI* transcatheter aortic valve implantation, *MAE* major adverse events, *PPMI* permanent pacemaker implantation after TAVI, *no-PPMI* patients without need for permanent pacemaker implantation after TAVI

Data for the outcome at one year are displayed in Tab. 4. One-year follow-up was obtained in 532 patients. All-cause mortality was 12.4%, 12.5% in no-PPMI patients and 12.2% in PPMI patients (*p* = 0.94) (Fig. 1a). The combined endpoint of major adverse events was not different between groups (22.1% no-PPMI vs. 24.5% PPMI, *p* = 0.55) (Fig. 1b). There was no relevant difference between groups with regard to the individual events of the combined endpoint, except for a trend towards a higher rate of transient ischaemic attack after PPMI in contrast to no-PPMI (4.8% vs. 1.8%, *p* = 0.06). Patients with PPMI after TAVI were significantly more often not admitted to a hospital due to cardiac symptoms compared with patients without PPMI (16.1% vs. 18.1%, *p* = 0.63). In patients with a LVEF below 45% (18.1% of the study population without prior PPMI) there was no impact of postprocedural PPMI on all-cause mortality (14.3% vs. 15.7%, *p* = 0.86). Even though the rate of the combined endpoint of major adverse events was numerically lower in patients with LVEF <45% and PPMI, it did not reach statistical significance (17.9% vs. 26.5%, *p* = 0.36).

**Table 4:** Clinical outcome at one year

	No-PPMI	PPMI	*P*-Value
Number of patients	385	147	
Major adverse events	22.1%	24.5%	0.55
– Only Boston Lotus valve – Only Edwards Sapien 3	24.3% 19.8%	19.4% 29.6%	0.43 0.12
All-cause mortality	12.5%	12.2%	0.94
– Only Boston Lotus Valve – Only Edwards Sapien 3	9.9% 14.3%	13.6% 11.3%	0.43 0.53
Stroke or transient ischaemic attack – Stroke – Transient ischaemic attack	5.5% 3.9% 1.8%	8.9% 4.1% 4.8%	0.15 0.91 0.06
Myocardial infarction	0.5%	0.7%	0.82
Bleeding	2.6%	2.7%	0.93
Aortic dissection	0%	0.7%	0.1
Aortic valve thrombosis	0.5%	0.7%	0.82
Endocarditis of the prosthetic aortic valve	0%	0.7%	0.10
New onset of atrial fibrillation	0.8%	0.7%	0.91
Rehospitalisation	18.1%	16.1%	0.63

**Fig. 2:**
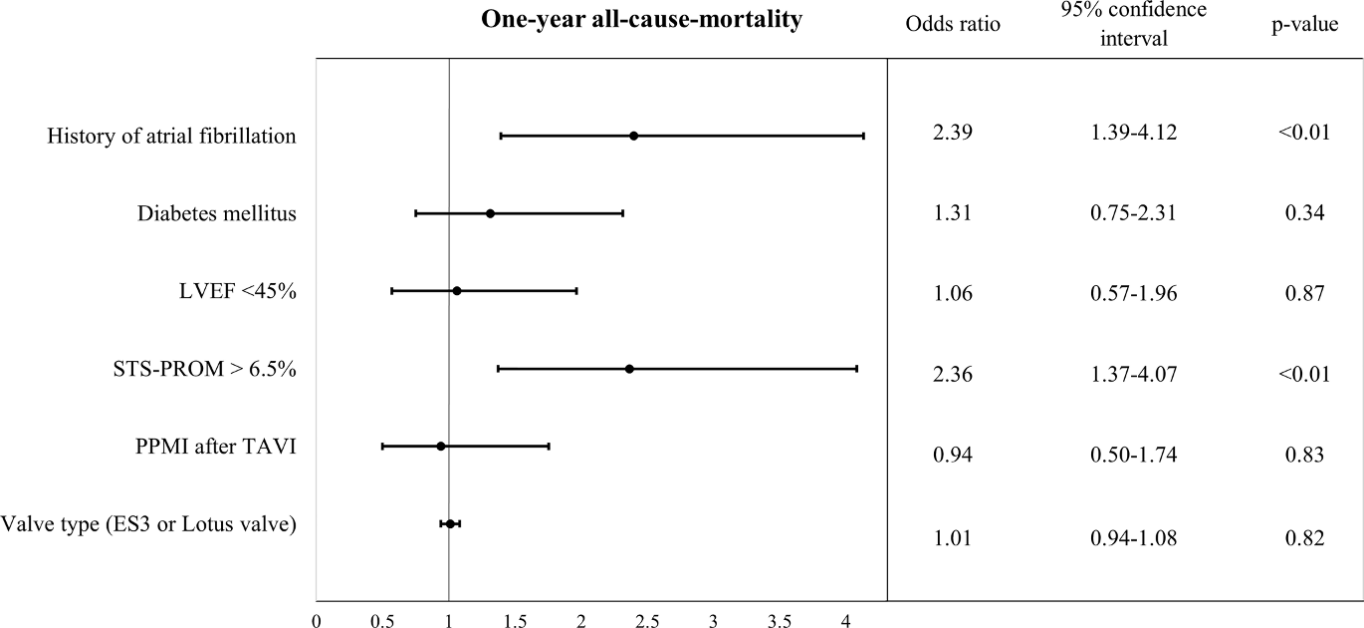
Odds ratios for one-year all-cause mortality. *LVEF* left ventricular ejection fraction, *PPMI* permanent pacemaker implantation, *TAVI* transcatheter aortic valve implantation, *STS-PROM* Society of Thoracic Surgeons Society for predicted risk of mortality

A subgroup analysis of patients with only one valve type (ES3 or Boston Lotus valve) did not reveal different outcomes concerning one-year mortality or rate of the combined endpoint.

### Multivariate logistic regression analysis

Multivariate logistic regression analysis including the parameters PPMI after TAVI, STS score for mortality >6.5%, LVEF <45%, diabetes mellitus, valve type (ES3 and Boston Lotus valve) and history of atrial fibrillation, revealed history of atrial fibrillation and STS-PROM >6.5% to be independently associated with one-year all-cause mortality. PPMI after TAVI, however, was not associated with one-year all-cause mortality (odds ratio [OR] 0.94, 95% confidence interval [CI] 0.50–1.74, *p* = 0.83; Fig. 2).

## Discussion

This study evaluates the impact of PPMI after TAVI on clinical outcome and mortality with new-generation TAVI devices. Our study with a PPMI rate of 24.4% did not reveal a worsened 30-day or one-year outcome for these patients with low total all-cause mortality rates of 1.3% and 12.4% respectively, even for patients with reduced LVEF. The low all-cause mortality rates we experienced with the new-generation devices are in line with recently published data on the ES3 with 1.9% and 11.8% respectively for a transfemoral access [20] and the Evolut R with survival rates of 99.0% and 85.8% for 30 days and one year [9].

However, there have been conflicting findings regarding the impact of PPMI on the outcome after TAVI with the early-generation devices. In the PARTNER trial the presence of a new permanent pacemaker was associated with higher one-year mortality (hazard ratio [HR] 1.38, 1.00 to 1.89, *p* = 0.05, 26.3% vs. 20.0%), as well as worsened clinical and echocardiographic outcomes for these patients in comparison with patients without pacemaker and left bundle branch block. They had significantly higher rates of rehospitalisation (42% vs 31.9%, *p* = 0.03), and significantly lower LVEF at one year after TAVI (55.4 ± 9.9% vs. 57.6 ± 8.2%, *p* = 0.01) [21]. However, in that same patient collective the impact of PPMI on mortality did not persist when compared with the whole population of patients without PPMI after TAVI, including those with left bundle branch block (one-year mortality 26.3% vs. 20.8%, *p* = 0.08) [22]. Recent data from the FRANCE 2 registry with 4,201 patients covering a follow-up of 3 years identified PPMI after TAVI with the self-expandable CoreValve and the balloon-expandable Edwards Sapien XT as an independent predictor for all-cause mortality. This stands in contrast to previous data from the FRANCE 2 registry (covering a shorter follow-up period of one year and only patients treated with the CoreValve device) concluding that PPMI did not have an impact on survival [12, 23]. Yet, there are several further studies that did not indicate PPMI to be independently associated with mortality after TAVI, including a meta-analysis of >20,000 patients. However, none of these studies covered the period of the latest data from the FRANCE 2 registry [24, 25]. Studies that demonstrated a negative effect on survival and occurrence of heart failure with PPMI included long periods of chronic right ventricular pacing [26, 27]. Therefore, the time periods for the impact of PPMI on the outcome of patients with TAVI that have been analysed up to now are most likely not long enough yet to reach clarity on this matter. Furthermore, there is evidence suggesting that pacing burden decreases in patients with PPMI after TAVI [28], a fact that we also experienced in our cohort, and there are data suggesting that worsening LVEF and higher rates of heart failure are related to higher ventricular pacing burdens [29]. Therefore, further studies evaluating these aspects on outcome will be necessary.

The rate of the combined endpoint of adverse events after hospital discharge in our study did not prove to be significantly higher for patients with PPMI. With the new-generation devices, we experienced low rates of cardiac and cerebral events. This is consistent with publications concerning the new-generation TAVI devices, especially the ES3 [20]. There is limited data on outcome >30 days with the Evolut R or Lotus valve. There seems to be no relation between these events and the presence of a pacemaker after TAVI.

In addition, the need for PPMI did not have an impact on rehospitalisation due to cardiac symptoms within 1 year after TAVI.

## Limitations

Like all registry data, this study in its design is prone to effects or possible confounders and bias. Furthermore, this is a single-centre study and therefore limited in its sample size.

## Conclusions

In this large all-comers TAVI population with new-generation devices the need for postprocedural PPMI did not show a statistically significant impact on survival or on combined endpoint of major adverse events within 12 months of follow-up.
